# PSA Secretion from Single Circulating Tumor Cells of Metastatic Castration-Naïve Prostate Cancer Patients

**DOI:** 10.1158/2767-9764.CRC-25-0158

**Published:** 2025-08-18

**Authors:** Eshwari Dathathri, Fikri Abali, Michiel Stevens, Richell Booijink, Tanja C. van Dijk, Khrystany T. Isebia, John W.M. Martens, Jaco Kraan, Nick Beije, Martijn P. Lolkema, Peter A.W. te Boekhorst, Paul Hamberg, Brigitte C.M. Haberkorn, Danny Houtsma, Joan van den Bosch, Wendy M. van der Deure, Ruchi Bansal, Leon W.M.M. Terstappen

**Affiliations:** 1 Department of Medical Cell Biophysics, Technical Medical Center, Faculty of Science and Technology, University of Twente, Enschede, the Netherlands.; 2 VYCAP, Enschede, the Netherlands.; 3 Department of Medical Oncology, Erasmus MC Cancer Institute, University Medical Center Rotterdam, Rotterdam, the Netherlands.; 4 Amgen, Thousand Oaks, California.; 5 Department of Hematology, Erasmus MC, University Medical Center Rotterdam, Rotterdam, the Netherlands.; 6 Department of Internal Medicine, Franciscus Gasthuis & Vlietland, Rotterdam/Schiedam, the Netherlands.; 7 Department of Medical Oncology, Maasstad Hospital, Rotterdam, the Netherlands.; 8 Department of Internal Medicine, Haga Hospital, The Hague, the Netherlands.; 9 Department of Internal Medicine, Albert Schweitzer Hospital, Dordrecht, the Netherlands.; 10 Department of Medical Oncology, Groene Hart Hospital, Gouda, the Netherlands.; 11 Department of General, Visceral and Pediatric Surgery, Heinrich-Heine University, University Hospital Düsseldorf, Düsseldorf, Germany.

## Abstract

PSA is the most common biomarker used in the screening and monitoring of prostate cancer. However, changes in PSA do not always reflect disease dynamics in every patient, and antihormonal agents may modulate its levels without significant antitumor effects. Changes in circulating tumor cells (CTC) have been described as a more objective measure of treatment response. Differences between PSA and CTC may be explained by heterogeneity in tumor cells producing PSA. To explore this, we measured the PSA secretion from a single CTC to gain insights into the PSA secretion heterogeneity between tumor cells. CTCs were enriched using EpCAM-based immunomagnetic enrichment in diagnostic leukapheresis of 18 patients with metastatic castration-naïve prostate cancer (mCNPC) not pretreated with any therapy, including androgen deprivation therapy. Calcein^+^ CD45^−^ cells were sorted by flow cytometry and deposited as single cells on a nanowell array to measure the PSA secretion after 24 hours. In nine of 18 patients, PSA secretion was detectable and observed from both prostate-specific membrane antigen–positive and prostate-specific membrane antigen–negative CTCs. In these patients, 29% to 100% (mean, 52; median, 47) of CTCs secreted PSA, with average PSA secretion levels ranging from 4 to 11.68 pg/cell (mean, 6.38 ± 2.29; median, 6.05). Notably, a strong heterogeneity in PSA secretion was observed within each patient. Our study demonstrates that CTC in mCNPC, even before therapy, produces varying amounts of PSA and often no PSA. These findings may explain the shortcomings of PSA as a biomarker for therapy response.

**Significance:**

This study reveals heterogeneity in PSA secretion among individual CTCs from patients with mCNPC prior to any therapeutic intervention, thereby highlighting the limitations of PSA as a biomarker.

## Introduction

Treatment options for metastatic castration-sensitive prostate cancer are rapidly expanding. In the last decade, we have learned that clinical outcomes improve when combining androgen deprivation therapy (ADT) with either chemotherapeutic agents, such as docetaxel (1), or with androgen receptor signaling inhibitors (ARSI), including apalutamide (2), enzalutamide (3), darolutamide (4), or a combination of them (5–7). Despite these advantages in treatment benefits, a clear heterogeneity in the treatment response of these patients is observed. Currently, there is an unmet need in predicting which patients will respond positively and who will have poor outcomes to a given treatment (8, 9). Clinicians rely on biomarkers such as PSA for monitoring treatment response. Despite high sensitivity, serum PSA levels do not always adequately reflect response to the treatment, with a significant number of patients not having PSA progression at the time of radiological progression (10, 11).

Liquid biopsies allow for real-time detection and analysis of circulating tumor cells (CTC), circulating nucleic acids, and extracellular vesicles that are constantly shed into the bloodstream from primary and metastatic tumor sites (12, 13). CTCs are well established for their clinical utility to provide not only valuable prognostic information that predicts treatment outcomes (14–17) but also reflects tumor composition and intra-patient heterogeneity (13, 18–20) that can be further investigated to improve the understanding of tumor progression.

Many studies have investigated CTC-based surface proteins as potential candidates for monitoring treatment response. For example, prostate-specific membrane antigen (PSMA) on CTCs in metastatic castration-resistant prostate cancer (CRPC) showed an association with poor treatment response and shorter overall survival, and recent studies indicate an inter- and intra-patient heterogeneity during treatment with abiraterone and enzalutamide (21, 22). However, no studies have investigated the utility of CTC-derived proteins such as PSA in patients with metastatic prostate cancer. As CTCs in circulation originate from primary and metastatic tumor sites, analyzing PSA from individual CTC can enhance the assessment of tumor heterogeneity and improve our understanding of PSA as a tumor marker. Currently, the portion of CTCs that secrete PSA and the amount of PSA they produce that contributes to serum PSA levels remain unknown. Information on PSA secretion at the individual cell level is only available from prostate cancer cell lines (23, 24). Preliminary studies on two patients with metastatic castration-naïve prostate cancer (mCNPC) indicated PSA secretions from CTCs and revealed heterogeneity (25). Based on the heterogeneity observed in PSA secretion from cell lines, PSA secretions from two patient samples, and the limitations of serum PSA to reflect treatment response, our research aim was to obtain insights into PSA secretion from individual CTC.

To overcome the rarity of CTC, we performed diagnostic leukapheresis (DLA) to enrich for CTCs on a unique cohort of patients with metastatic castration-sensitive prostate cancer before they received ADT for *de novo* metastatic disease, which is true mCNPC. We utilized immunomagnetic EpCAM-based enrichment, followed by FACS of calcein^+^ CD45^−^ cells and seeding as single cells on nanowell arrays (26) to probe for single-cell PSA secretion and PSMA expression.

## Materials and Methods

### Patient samples

In the PICTURES (trial ID: NL8549) study, 160 mCNPC were included before starting ADT between September 2020 and August 2023. Patients were screened for the presence of CTCs and underwent a DLA subsequently if their CellSearch CTC count was three or higher in 7.5 mL of blood. Of the 160 patients screened, 56 were eligible and 38 of the 56 consented and successfully underwent a DLA procedure. The procedure to measure PSA secretion of CTC was investigated and optimized on the first 20 DLAs and then successfully executed on DLAs from 18 patients. The study participants signed informed consent before any study procedures. This study was performed in agreement with the Helsinki Declaration, and the protocol of the PICTURES study (MEC 20-0422) was approved by the involved Medical Research Ethics Committees. The characteristics of the 18 patients included in this study are provided in Table 1.

**Table 1:** Baseline characteristics

Characteristic	*N* = 18
Age at registration	​
Median (range), years	74 (58–79)
WHO PS at registration, *n* (%)	​
0	10 (55.6%)
1	8 (44.4%)
Initial PSA, *μ*g/L	​
Median (range)	128.3 (15.1–4,682)
Hemoglobin, mmol/L	​
Median (range)	8 (5.7–9.9)
Alkaline phosphatase, IU/L	​
Median (range)	234.5 (83–2,431)
Lactate dehydrogenase, IU/L	​
Median (range)	224.5 (177–644)
Gleason score by diagnosis	​
Median (range)	7 (6–10)
M-stage at diagnosis	​
M1a	0
M1b	14 (77.8%)
M1c	2 (11.1%)

Abbreviation: WHO PS, World Health Organization performance score.

Range = min–max. Patients with mCNPC with missing information: alkaline phosphatase (*n* = 2), lactate dehydrogenase (*n* = 2), and patients presenting with lymph node (N1) metastasis (*n* = 2).

### The DLA procedure

Patients with ≥3 CTCs per 7.5 mL of blood as assessed by CellSearch underwent the DLA procedure at the Department of Hematology at the Erasmus Medical Center using the Spectra Optia Cell Separator machine (Terumo BCT). A maximum volume of 6L of peripheral circulating blood was processed and citrate dextrose solution A was used as an anticoagulant. For collection, the standard settings for white blood cell (WBC) isolation were applied according to previously conducted studies (20); only the plasma rate was increased to collect a slightly higher cell density. A blood volume of 5,000 to 6,000 mL (mean, 5,295 mL; median, 5,041 mL) was processed and a volume of 76 to 116 mL (mean, 90 mL; median, 91 mL) of DLA product containing 40.19 to 175.70 × 10^6^ WBCs/mL (mean, 98 × 10^6^ WBCs/mL; median, 84 × 10^6^ WBCs/mL) was obtained from 18 patients.

### Cell line and cell culture

LNCaP (CRL-1740, RRID: CVCL_0395) and PC3 (CRL-1435, RRID: CVCL_0035) cells obtained from the ATCC were cultured in a complete medium made of RPMI-1640 medium (Lonza) supplemented with 10% FBS (Sigma-Aldrich) and 100 U/mL penicillin and 100 μg/mL streptomycin (Lonza). All the cell lines were cultured in T25 treated culture flasks (VWR international B.V) at a cell density of 5,000 cells/cm^2^ in a humidified incubator at 37°C and 5% CO_2_. To subculture, the LNCaP and PC3 cells were first washed with PBS (Sigma-Aldrich). Pre-warmed 0.05% trypsin–EDTA was added to the cells, followed by incubation for 1 minute at 37°C. After the cells detached, they were collected in fresh medium with pipetting and counted using the Luna-FL automated cell counter (Westburg B.V.). The cells of desired concentration were transferred into new culture flasks and maintained in a humidified incubator at 37°C and 5% CO_2_. The RWPE-1 (CRL-3607, RRID: CVCL_3791) cells obtained from ATCC were cultured in keratinocyte serum-free medium supplemented with human EGF (5 ng/mL) and bovine pituitary extract (0.05 mg/mL; ATCC). The cells were washed with Dulbecco’s PBS (DPBS; Lonza), followed by trypsinization with a 1:1 dilution of 0.05% trypsin–EDTA (Gibco, Thermo Fisher Scientific) in DPBS and incubated for at least 2 minutes. The cells were then collected in DPBS + 2% FBS (Sigma-Aldrich) to deactivate the trypsin. The desired cell concentration was added to a new culture flask with fresh cell culture medium. The cell culture medium for all cell lines was changed every 2 days and the cells were harvested when they reached 70% to 80% confluency. CTC cell suspension for sorting and seeding was prepared in the adjusted prostate cancer organoid medium as previously described (20), supplemented with recombinant R-spondin1 and Noggin (Sigma-Aldrich) with concentrations as previously described (27), and with 5 nmol/L of androgen stimulator R1881 (Biotang Inc.). The components of the adjusted prostate cancer organoid medium are described in Supplementary Table S1. All cell lines used in this study were tested regularly for the absence of *Mycoplasma* contamination using standard PCR assays and were authenticated by short tandem repeat analysis.

### Enumeration of CTC using CellSearch and ACCEPT

To obtain a reference for the CTC counts, DLA aliquots of 200 × 10^6^ cells collected in CellSave preservative were enriched with the CellSearch CXC kit on the CellTracks Autoprep (Menarini Silicon Biosystems). The DLA samples were placed in a conical tube, supplemented with the dilution buffer (total volume 11 mL), and centrifuged at 400 *g* for 10 minutes. Subsequent processing with the CellSearch system included EpCAM ferrofluids for immunomagnetic enrichment of CTC and staining with the fluorescent reagents 4′,6-diamidino-2-phenylindole (DAPI), cytokeratin (CK) 8, 18, 19-phycoerythrin, and leukocyte-specific CD45-allophycocyanin (CD45-APC). A 5.7 μg/mL concentration of anti–human PSMA (FOLH1) PE clone LNI-17 (cat. #342504, RRID: AB_2247193, BioLegend) was added to the reagent cocktail. After enrichment and staining, the CellSearch cartridge within the CellTracks magnet holder was placed on the CellTracks Analyzer II (Menarini Silicon Biosystems) for image acquisition using the default settings. The digitally stored fluorescence image files were analyzed with the open-source ACCEPT software v1.1 (http://github.com/LeonieZ/ACCEPT) using the “Full Detection” function.

### Enrichment of CTC from DLA products for functional analysis

Immediately after leukapheresis, the CTC counts were assessed using the standard CellSearch system. The DLA products were placed in 10 mL EDTA tubes at concentrations ranging from 200 to 2,000 × 10^6^ WBCs and shipped to the Medical Cell BioPhysics laboratory of the University of Twente. The DLA samples were processed immediately upon arrival (within 26 hours after leukapheresis). Immunomagnetic enrichment targeting EpCAM was performed with the reduced enrichment reagent protocol (28). Briefly, the DLA product was split into 1 mL aliquots and incubated with 15 μL anti–EpCAM-conjugated ferrofluids (CellSearch) each for 10 minutes on a magnet (iMag, Becton Dickinson). Next, 15 μL of capture enhancement mix (CellSearch) was added and incubated twice for 10 minutes on the magnet. The samples were mixed in between the two incubation steps. Subsequently, 2 mL buffer [PBS supplemented with BSA (Merck), EDTA (Merck), casein (Merck), and mouse serum (Invitrogen)] was added, mixed, and incubated for 20 minutes on the magnet for magnetic separation. The unbound fraction was gently aspirated with a glass Pasteur pipette using a syringe pump at 1 mL/minute speed. The samples were then washed in 1 mL buffer for 10 minutes on the magnet and the unbound fraction was removed with a syringe pump, leaving behind the enriched sample.

### Preparation of nanowell arrays for cell seeding

Nanowell arrays (VyCAP B.V) containing 6,400 nanowells were sterilized in 70% ethanol. To remove any ethanol and air in the nanowells, the array was then washed by placing it into a desiccator, and vacuum pressure (−0.5 bar) was applied to allow sterile PBS to enter the wells. Subsequently, the nanowell array was inserted in a filtration holder and placed in a pump unit (VyCAP). A pressure of 5 to 10 mBar was applied to wash the chip with 5 mL of PBS. Subsequently, the nanowell was washed and incubated with a complete culture medium before seeding the cells.

### Preparation of the membranes for PSA detection

A low-fluorescence polyvinylidene membrane (PVDF) with 0.45 µm pore size (Bio-Rad) was cut to 1 × 1 cm dimensions and activated using 100% methanol (Thermo Fisher Scientific), followed by three washes in sterile Milli-Q. Next, the membranes were coated with mouse anti-PSA (25 μg/mL; cat. #10-P21A, RRID: AB_1288494, Fitzgerald Industries International) overnight at 4°C. Subsequently, the membranes were washed in PBS (EMD Millipore) and blocked in 3% BSA (Sigma-Aldrich) in PBS for 1 hour at room temperature. Finally, the membranes were washed once in PBS and incubated in the CTC culture medium before use (29, 30).

### Staining, sorting, seeding, and imaging of CTC in nanowells

After the EpCAM-based enrichment (28), the enriched cells were resuspended in 100 μL of staining cocktail containing 0.5 μmol/L of calcein AM stain (cat. #C1430, Invitrogen), 1 μg/mL of anti–PSMA-PE (FOLH1) clone LNI-17 (cat. #342504, RRID: AB_2247193, BioLegend), and 4 μg/mL of anti–CD45-APC clone HI30 (cat. #20-0459-T500, Tonbo Biosciences, Thermo Fisher Scientific) for 30 minutes at 37°C. The cells were washed twice with 1% BSA in PBS for 5 minutes each using the BD magnet and aspirated with the syringe pump. The cells were resuspended in 500 μL in a complete CTC medium before FACS sorting of CTCs. A flow cytometer (FACSAria II BD Biosciences) was used to identify and sort the viable (calcein-FITC positive, CD45-APC negative) cell population into 12 × 12 mm tubes containing 200 μL of complete medium. Next, the cell suspension was added dropwise to the nanowell array, and a small negative pressure of 5 to 10 mbar was applied to pass the sample through (23, 29). Next, the nanowell array was removed from the filtration holder and placed in the Puncher system (VyCAP B.V), which is integrated with the inverted fluorescence Nikon TiE-2 microscope. The nanowells were imaged using the LED Sola Lumencor Light source, a 20X, NA 0.45 objective (Nikon), and a CMOS camera. Then 10 × 10 images of the entire 8 × 8 mm surface of the nanowell array using the filter cubes of brightfield, reflection, PE, FITC, APC, and BV421 at exposure times 50, 25, 200, 200, 200, and 200 ms, respectively, were acquired. The nanowell array was removed from the Puncher system and inserted into the Clamp unit (VyCAP B.V). The prepared PVDF PSA-capturing membrane was then sandwiched between the bottom of the nanowell array and a PDMS slab present in the clamping unit. The anti–mouse IgG added to the medium diffuses through all the pores of the chip ensuring an imprint of the nanowells on the membrane needed for the correlation of cells (on the array) with the corresponding secretion spots (on the membrane). Finally, the clamping unit was incubated at 37°C and 5% CO_2_ for 24 hours.

### Detection of printed antibody arrays and imaging of membranes

To detect the PSA secreted after the overnight incubation, the PVDF membranes were carefully removed from the clamping unit. The membranes were washed once in PBS with 0.05% Tween 20 in PBS for 5 minutes to remove cell debris. Next, the membranes containing the captured PSA were incubated with a solution of 2 μg/mL of rabbit anti-PSA (cat. #Ab75684, Abcam, stock 8.75 mg/mL) prepared in 1% BSA in PBS at room temperature for 1 hour on the thermoshaker at 300 rpm. The membranes were washed thrice for 5 minutes with 1% BSA in PBS. The membranes were incubated with a solution of 2 μg/mL of goat anti–rabbit IgG Alexa Fluor 488 (Abcam, cat. #150077, RRID: AB_2630356, stock 2 mg/mL) prepared in 1% BSA in PBS for 1 hour at room temperature on the thermoshaker at 300 rpm. Finally, the membranes were briefly washed twice in 1% BSA in PSA and once in Milli-Q for 5 minutes each and then air dried. The membranes were imaged with the Puncher system (VyCAP B.V) in the FITC and PE channels at exposure times of 100 and 150 ms, respectively, to visualize the PSA spots and the imprints of the nanowells.

### Image analysis and quantification of fluorescent signal

To identify secretions from their respective cells and quantify these secretions, the SPOT software (VyCAP B.V) developed using the LabVIEW’s IMAQ image analysis routines (National Instruments) was used (26). Using the SPOT software, the imprint developed using the PE channel was first loaded to identify and match the position of the membrane image with the chip scan. The edges of the imprint were identified and saved as they correlate the boundaries of the chip. Next, the image containing the PSA spots (imaged in FITC) was loaded. Using the edges saved from the imprint, the location of the PSA spots on the FITC-imaged membrane was correlated to the well numbers of the nanowell array. This allowed the PSA secretion to be traced to the corresponding nanowell number. The intensities of the PSA secretion along with the nanowell numbers with the cells of interest were extracted.

### PSA calibration curve

The calibration curves were prepared by spotting the PVDF membranes with 2 μL of PSA peptide (cat. #ab41421, Abcam) prepared in dilutions ranging from 31.5 to 500 μg/mL. The membranes were blocked and stained with the antibodies as described in section 2.9 and the average fluorescence intensity of the spots was determined using the ImageJ (version 1.54) software. By measuring the spot size of the spotted medium with PSA, the amount of PSA in pg/μm^2^ was next calculated by dividing the amount of PSA present in the 2 μL drop by the area of the spot. The measured intensity versus the different amounts of PSA in pg/μm^2^ was plotted. Using these calibration values, the amount of PSA in pg/μm^2^/cell was determined using the PSA spot intensities from CTCs extracted from the membranes. To obtain the area of the PSA spots captured on the membranes, the membranes were analyzed using ImageJ using the threshold function to extract the area of the spots. The PSA in pg/cell was calculated by multiplying the area values with the corresponding PSA values in pg/μm^2^/cell.

### Statistical analysis

Statistical analysis such as the two-tailed Spearman ρ test was performed to evaluate the relationship between the CK-identified and PSMA-identified CTCs of patients with mCNPC using the OriginPro software (v2021b, Origin Lab Corporation). Graphs were plotted using OriginPro and Python 3.9.1.

### Data availability

The data generated in this study are available in the article and its supplementary data files.

## Results

### Enumeration of CTC based on CK and PSMA

CTCs identified by the CellSearch system were immunomagnetically enriched by targeting EpCAM and identified by CK^+^ expression, lack of CD45 (CD45^−^), presence of a nucleus (DAPI^+^), and morphology associated with a cell and were verified by a trained observer. As CK is an intracellular protein, it cannot be identified without loss of cell viability, thus preventing its use for our planned functional (single-cell PSA secretion) studies. As an alternative, the cell surface protein PSMA was evaluated to identify CTC. To evaluate the concordance in the expression between CK and PSMA, a DLA aliquot of 200 × 10^6^ cells collected in a CellSave tube (fixed) was enriched using the CellSearch CXC kit on the AutoPrep, using PSMA-PE as an additional staining reagent. The images obtained from the CellTracks Analyzer II were analyzed with the ACCEPT software to extract CTCs that were DAPI positive, CD45−, and belonged to either group (i) CK^+^ PSMA^+^; (II) CK^+^ PSMA^−^; or (iii) CK^−^ PSMA^+^. The mean intensity threshold for CK (intensity ≥ 50) and PSMA (intensity ≥ 200) positivity was set using the prostate cell lines LNCAP, PC3, and RWPE-1 (Supplementary Fig. S1).

**Figure 1:**
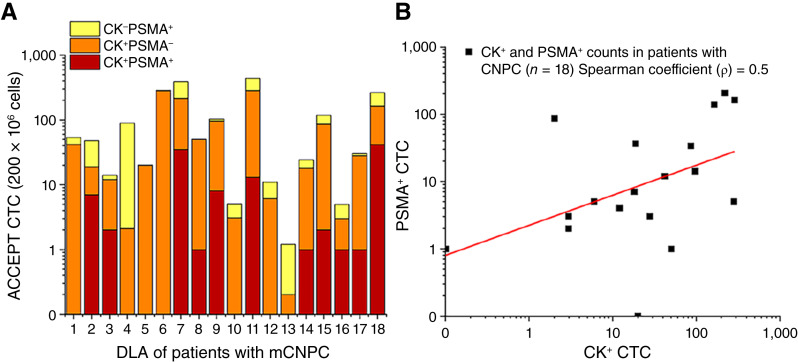
Enumeration of CTCs based on CK and PSMA expression. **A,** Graph depicting the number of DAPI^+^ CD45^−^ CTCs as the following groups: CK^−^PSMA^+^ (yellow), CK^+^ PSMA^−^(orange), and CK^+^ PSMA^+^ (red) in the DLA aliquot of the 18 patients. **B,** Correlation of the number of CK^+^ and PSMA^+^ CTCs (Spearman coefficient ρ = 0.5).

Figure 1 shows the enumeration of the different CTC groups from the DLA aliquots of the 18 patients. Not all CK^+^ CTCs expressed PSMA above the threshold and not all PSMA^+^ CTCs expressed CK. The regression analysis between CK^+^ and PSMA^+^ CTCs was log_10_ (PSMA^+^ CTC) = (0.45 ± 0.22) × log_10_ (CK^+^ CTC) + (0.35 ± 0.35) and showed a positive correlation with Spearman ρ coefficient = 0.5 (Fig. 1B). The number of CK^+^ CTCs (mean, 74.22 ± 96.90; median, 24) was higher than PSMA^+^ CTCs (mean, 40.33 ± 64.68; median, 6).

### Processing DLA of patients with mCNPC to obtain a single viable CTC

**Figure 2:**
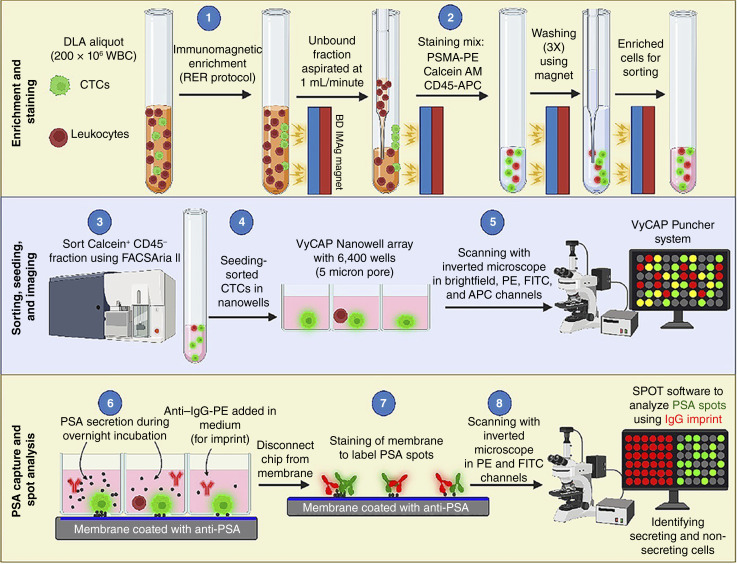
Workflow schematically illustrating the DLA processing to obtain viable CTCs. (1) DLA enrichment using the reduced enrichment reagent (RER) protocol; (2) staining using anti–PSMA-PE, anti–CD45-APC, and viability dye calcein AM; (3) sorting of calcein^+^ and CD45^–^ CTCs enriched from the DLA; (4) seeding into the VyCAP nanowells; (5) imaging using a fluorescence inverted scanning microscope to visualize cells captured in the nanowells; (6) PSA secretion from single cells and PSA capture overnight (24 hours) using an anti–PSA-coated membrane and addition of anti–IgG-PE to visualize the imprint; (7) staining of the membrane for visualization of PSA secretion spots; and (8) correlation of PSA spots with cells with the help of imprint and the SPOT software to identify PSA-secreting and non-secreting PSMA^+^/PSMA^−^ CTCs.

The workflow outlined in Fig. 2 was used to process 18 patient DLA samples. Because of the positive correlation between PSMA and CK, the staining mix was adjusted to include antibodies targeting PSMA instead of CK. The processing of the DLA aliquots followed three major steps: enrichment and staining, sorting, seeding, and imaging, and finally, PSA capture and spot analysis.

#### CTC enrichment, sorting, and single-cell isolation

**Figure 3:**
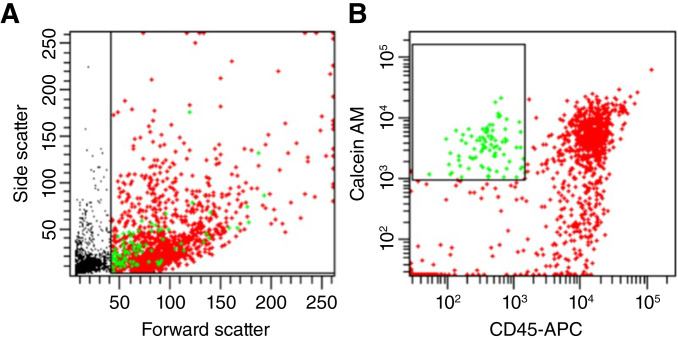
Flow cytometry analysis of EpCAM-enriched DLA sample. **A,** Flow plot showing the forward and side scatter of the sample with a gate excluding small events (in black). **B,** CD45-APC vs. calcein AM scatter plot of the events with a gate on the calcein^+^ CD45^−^ cells (in green) with all other events (in red).

DLA aliquots (ranging from 200 × 10^6^ to 800 × 10^6^ cells) were immunomagnetically enriched by targeting EpCAM [Fig. 2 (step 1)] using the reduced enrichment reagent protocol (28). The enriched samples were stained with calcein AM (a viability dye), PSMA-PE, and CD45-APC [Fig. 2, (step 2)], followed by FACS sorting of calcein^+^ CD45^−^ events and were collected in 200 μL of the CTC medium [Fig. 2 (step 3)]. Figure 3 shows an example flow plot and the gating strategy used to sort cells from the EpCAM-enriched DLA aliquot. The gating was performed to exclude the small events (depicted in black) as shown in Panel A. From the big events, the tumor cells were then identified by plotting calcein AM against CD45-APC and gating the population that is positive for calcein and negative for CD45 expression (depicted in green) in Panel B. The green population was then sorted for single-cell isolation and PSA capture.

The number of sorted calcein^+^ CD45^−^ cells in the 18 DLA samples ranged from 46 to 7,046, (mean, 1,636; median, 1,218, SD 1,818). The DLA-sorted samples were seeded onto the nanowell array using a gentle negative pressure of 5 mbar [Fig. 2 (step 4)]. As the single-cell suspension flows through the nanowells, individual cells either pass through or block the pore at the bottom, preventing the flow and resulting in cell entrapment. After seeding, the chip was examined for the presence of cells by fluorescence microscopy [Fig. 2 (step 5)]. The number of cells detected in the nanowell chip ranged from 0 to 1756 (mean, 121; median, 5; SD, 412), resulting in a recovery rate of 0% to 25% (mean, 4; median, 1; SD, 7). In three patient samples, no CTCs were detected in the nanowell array.

The low yield can be attributed to several factors. Some DLA samples contained small CTCs that passed through the nanowell pores, and the living cells were more likely to pass through the pores than fixed cells. Additionally, most DLAs contained substantial cellular debris. Although sorting helped to remove much of the debris (Supplementary Fig. S2), the cellular debris (with nonspecific staining for calcein) clogging the microwells was still observed, further reducing the yield. This issue was compounded by the fact that many sorted events likely represented cellular debris, of which CTCs were possibly only a small subpopulation.

**Figure 4:**
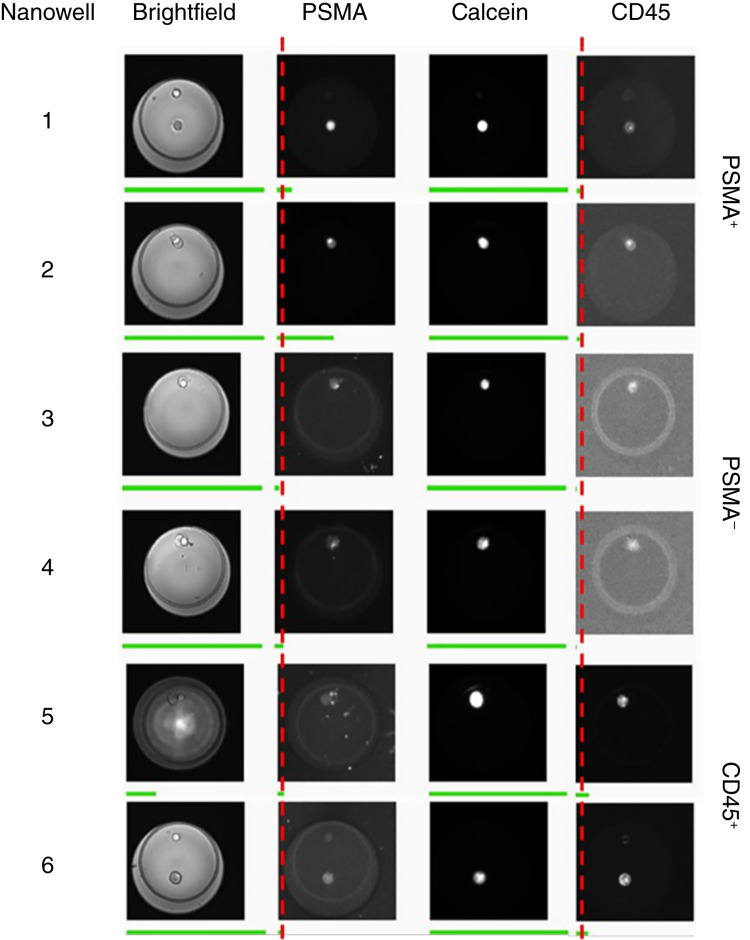
Single-cell seeding of CTCs in a nanowell chip. Images of nanowells after seeding of sorted DLA samples. Nanowells 1 and 2 show calcein^+^ PSMA^+^ CD45^−^ cells and nanowells 3 and 4 show calcein^+^ PSMA^−^ CD45^−^ cells. The green bar provides an estimate of the intensity levels per image. The cells that are stained brightly are viewed as positive. The red dashed line is set at the edge of the green bar of thumbnails 3 and 4. PSMA intensity (green bar) beyond this threshold indicates a positive signal as seen in the nanowells 1 and 2. CD45 intensity beyond this threshold indicates a positive signal and the presence of leukocytes in nanowells 5 and 6.

Of the 15 samples in which the cells were captured on the chip, the percentage of cells expressing PSMA ranged from 0% to 100 % (mean, 72; median, 94; SD, 39) whereas the percentage of cells expressing no PSMA ranged from 0% to 100% (mean, 28; median, 6; SD, 39). Fig. 4 shows examples of calcein^+^ PSMA^+^ CD45^−^ cells and calcein^+^ PSMA^−^ CD45^−^ cells captured in the nanowells after sorting and seeding.

#### Single CTC PSA detection

**Figure 5:**
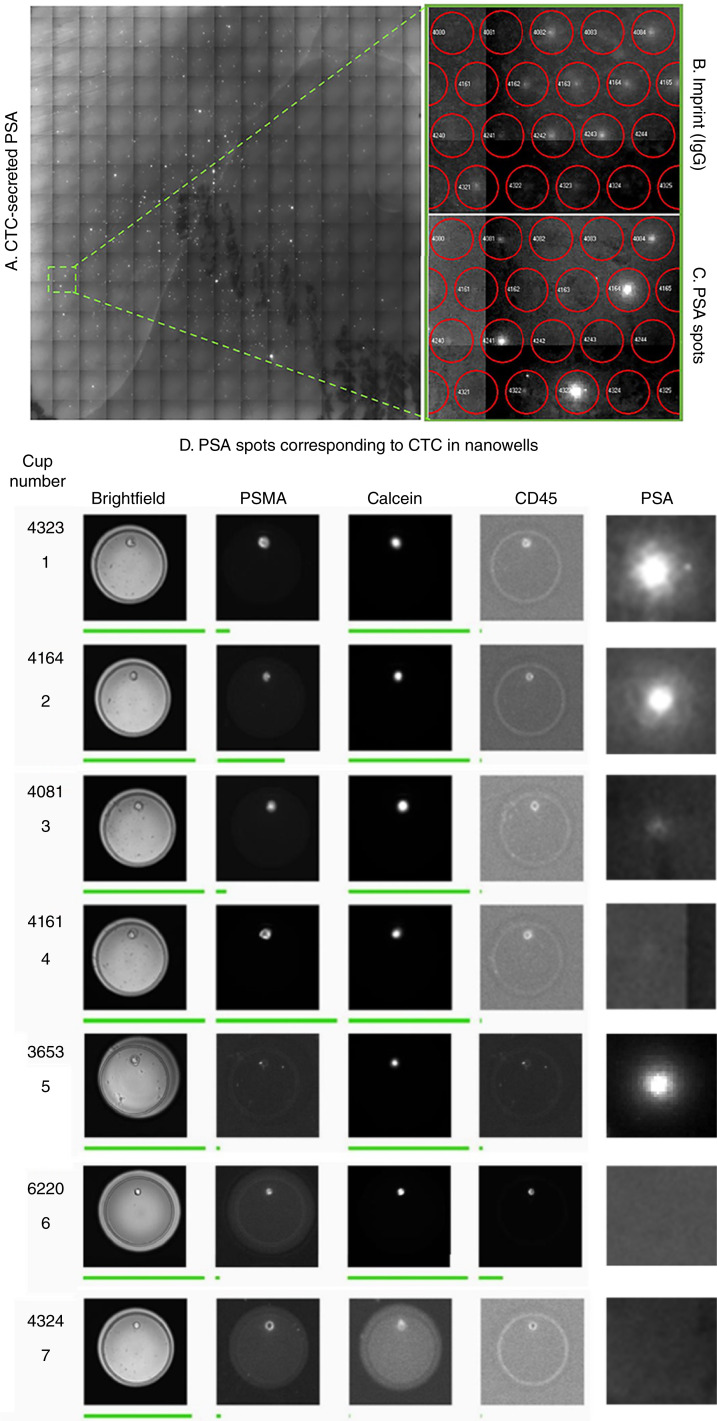
Measurement of PSA secretion of individual CTC captured on a membrane placed below the nanowell array. **A,** The image of the membrane detached from the nanowell array and stained with PSA-FITC and printed with anti–IgG-PE. The squares are the individual images obtained with the FITC filter cube, and the white spots represent the secreted PSA identified through FITC staining. The position on the membrane indicated with dashed green square is magnified to visualize the imprint of the chip and the PSA secretion on the membrane. **B,** The PE channel read out of the membrane that the SPOT software used to align the wells (red circles) on the membrane and the numbers indicate the well numbers of the nanowell. **C,** The corresponding PSA signal in the FITC channel of the respective well. **D,** Brightfield, PSMA, calcein, and CD45 images of seven nanowells with the corresponding secreted PSA on the membrane. Nanowells 1, 2, 3, and 4 show PSMA+ CTC secreting high, medium, low, and no PSA, respectively. Nanowell 5 shows a well with a PSMA^−^ CTC secreting PSA. Nanowell 6 shows a PSMA^−^ calcein^+^ cell with no secretion and nanowell 7 shows a well with no cell and no secretion. The green line below the nanowell images indicates the intensity of the fluorescent signal detected across the image.

To match the position of the nanowells to the spots on the membrane representing PSA secretion, an imprint of the chip on the membrane is necessary. This was achieved by flowing anti–IgG-PE through the pores of the wells of the chip and onto the membrane, which helps to develop an imprint of the wells on the membrane, as illustrated in Fig. 2, step 6 and 7. The imprint developed is used to align the position of the wells of the chip to the spots on the membrane using the SPOT software [Fig. 2 (step 8)]. The spots of PSA secretion of single CTC can now be identified, as shown in Fig. 5. A detailed overview of the membrane visualized for imprint (in PE) and PSA spots (in FITC) and their corresponding locations on the chip visualized for brightfield, calcein (in FITC), PSMA (PE), and CD45 (in APC) is shown in the Supplementary Fig. S3.

**Figure 6:**
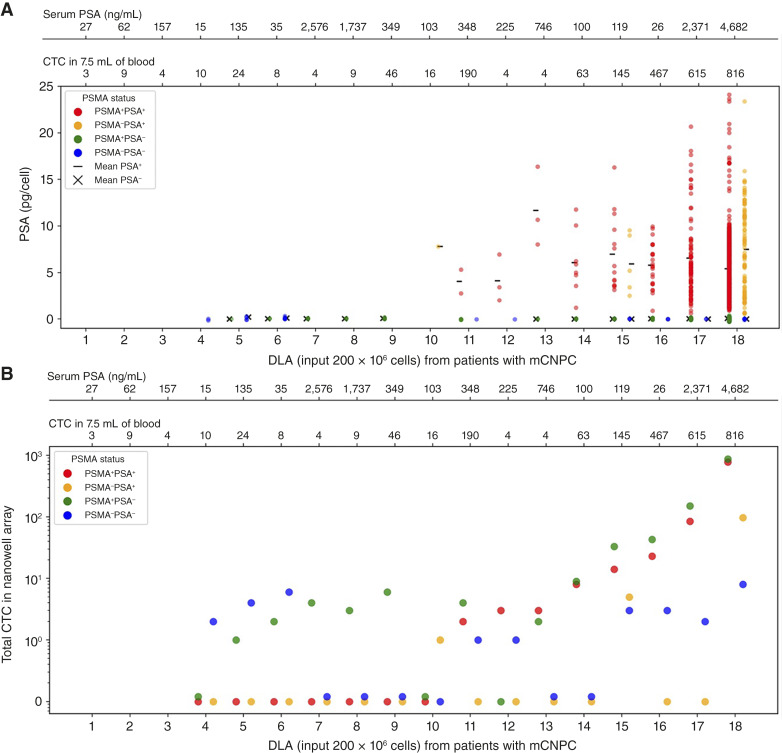
PSA secretions (pg/cell) from single CTC and total CTC captured in nanowell array from patients with mCNPC (*n* = 18). **A,** The PSMA^+^ PSA^+^ (in red) and PSMA^−^PSA^+^ (in yellow) indicate the two subpopulations of CTC that secrete. The PSMA^+^ PSA^−^ (in green) and PSMA^−^PSA^−^ (in blue) indicate the two subpopulations of CTC that are non-secreting cells. The serum PSA (ng/mL) and the CTC in 7.5 mL of blood are indicated on the secondary *x*-axis. **B,** Total counts of the CTC captured in the nanowell array indicating PSMA^+^ PSA^+^ (in red), PSMA^−^PSA^+^ (in yellow), PSMA^+^ PSA^−^ (in green), and PSMA^−^PSA^−^ (in blue).

The PSA secretion detected on the membrane and thus secreted from the individual cells in the nanowells from 18 patients is illustrated in Fig. 6. All mean intensities obtained from the PSA spots were normalized to the average of the combined membrane intensities corresponding to leukocytes and the no-cell wells (*n* = 50), thereby accounting for background membrane intensity or nonspecific staining on the membranes. Using a calibration curve generated from the serial dilutions of the PSA protein (Supplementary Fig. S4A and S4B), the PSA (in pg/μm^2^) was calculated using the respective intensities. The areas of the respective spots were calculated as shown in Supplementary Fig. S5 and used to obtain the PSA secretion values (in pg/cell).

The quantity of PSA in a single nanowell secreted by a calcein^+^ PSMA^+^ CD45^−^ cell is depicted in red and a cell that did not secrete PSA in green. The quantity of PSA from nanowells with calcein^+^, PSMA^−^, and CD45^−^ cells is depicted in orange and the cells that did not in blue. Above the figure, the serum PSA (ng/mL) and the number of CTCs (per 7.5 mL of blood) at the screening time are indicated. The patients are sorted according to (i) the presence of cells in the nanowells; (ii) whether or not PSA secretion was measured; and (iii) by increasing order of PSA-secreting cells in the nanowells.

In DLAs 1 to 3, zero CTCs were captured in the nanowells. In DLA 4, two CTCs were detected, both of which were PSMA^−^ with no detectable PSA secretion. In DLAs 5 and 6, PSMA^−^ CTCs were captured, but the cells did not secrete measurable PSA, whereas DLA 7 to 9 showed the presence of PSMA^+^ and PSMA^−^ CTCs that did not secrete PSA. In DLAs 10 to 18, PSA secretions were observed from PSMA^+^ and PSMA^−^ cells. The overview of the CTC recovery from the FACS sorting and seeding on the nanowells is provided in Supplementary Table S2.

In nine of 18 patients (DLAs 10–18), the percentage of PSA-secreting CTCs ranged from 29% to 100% (mean, 52; median, 47). The average PSA secretion ranged from 4 to 11.68 pg/cell (mean, 6.38 ± 2.29; median, 6.05). A notable heterogeneity in PSA secretion was observed both between individual CTC and across patients.

When looking at the screening CTC numbers and serum PSA values (acquired before the DLA) for inclusion into the study, a clear relation between increasing CTC number and serum PSA from left to right in the figure is discerned, suggesting an increasing chance of measurable PSA secretion of individual CTC with increasing CTC number and increasing serum PSA. Some exceptions are DLA 11 in which the high CTC number and serum PSA values did not translate into CTC capture and PSA production and DLA 16 in which high PSA secretion was observed despite having low serum PSA. In nine of the 18 patients, with PSA-secreting cells, a weak but positive correlation was observed between the serum PSA and CTC PSA with Spearman coefficient ρ = 0.28 (Supplementary Fig. S6).

**Figure 7:**
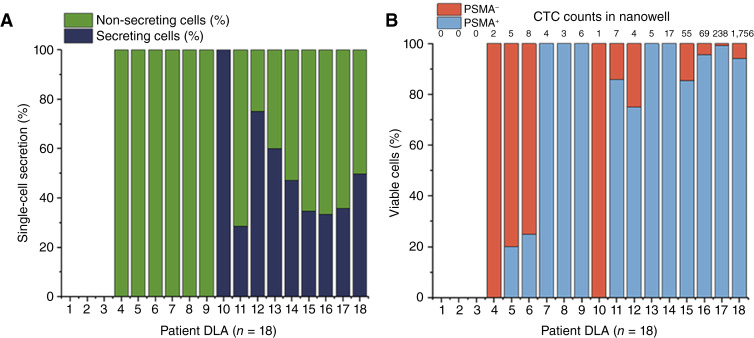
Distribution of CTC groups in the nanowells. **A,** Percentage of PSA-secreting and non-secreting cells detected in the nanowells and analyzed using the SPOT software. **B,** Percentage of PSMA^+^ and PSMA^−^ cells identified in the nanowells. There were 0 CTC captured in the nanowells of the first three DLA. The total CTC counts in the nanowells are represented for each patient above the columns.

The distribution of PSA-secreting and non-secreting cells, arranged in the same order as Fig. 6, is indicated in Fig. 7A. For 15 patients with mCNPC (excluding the first three DLAs in which 0 CTCs were found), the percentage of PSA-secreting cells ranged from 0% to 100% (mean, 31; median, 32). The percentage of PSA non-secreting cells ranged from 0% to 100% (mean, 69; median, 67), indicating that the majority of the CTCs do not secrete PSA. The distribution of the PSMA^+^ and PSMA^−^ cells is shown in Fig. 7B. For 15 patients with mCNPC, the percentage of PSMA^+^ cells ranged from 0% to 100% (mean, 72; median, 94), whereas PSMA^−^ cells ranged from 0% to 100% (mean, 28; median, 6). Among PSMA^+^ cells, the percentage that secretes PSA ranged from 0% to 75% (mean, 23; median, 25), whereas the PSMA^−^ cells that secrete PSA ranged from 0% to 100% (mean, 8; median, 0). Notable is that PSMA^−^ cells that secrete PSA can be considered as CTCs; however, for the PSMA^−^ cells that do not secrete PSA, we lack evidence to confirm that these cells are indeed CTCs.

## Discussion

Resistance to therapies is the greatest challenge observed in patients with metastatic prostate cancer given the very heterogeneous nature of the cancer cells. PSA is a protein made by both normal and cancerous prostate cells and is widely used to screen for prostate cancer and monitor treatment response (31, 32). PSA production is mainly controlled by the androgen receptor (AR), which responds to male hormones like testosterone. In early prostate cancer, androgens bind to AR, activating PSA gene expression—a process well understood and central to disease development (33–35). However, in advanced prostate cancer, especially CRPC, PSA levels often stay high despite treatments that lower hormone levels. This means that AR can stay active without androgens through mechanisms like gene amplification, mutations, alternative AR forms, or interactions with other signaling pathways. These changes help cancer cells bypass normal hormone controls and keep growing (35–37). Although most patients with metastatic prostate cancer show elevated PSA serum levels, these are not always adequate in reflecting tumor burden and have pitfalls in evaluating treatment response. The PSA-based metric that shows prognostic value in mCNPC is an undetectable PSA at 6 to 9 months, whereas other PSA-based makers fall short. For PSA to serve as a meaningful biomarker in the mCNPC setting, complete suppression of PSA production is required. Even trace amounts of PSA can indicate residual disease and reflect heterogeneity in PSA production (38–40). As CTCs are constantly shed into the bloodstream during tumor progression and growth, they have gained a lot of attention because of their ease of accessibility in peripheral blood and may be used as clinical biomarkers in addition to PSA in measuring treatment response. In this study, we explored whether heterogeneity in PSA secretion among tumor cells could contribute to the limitations of PSA as a biomarker. We demonstrate, for the first time, that PSA secretion can be measured at the single tumor cell level. Our findings reveal heterogeneity in secretion, with most CTCs not secreting PSA, in castration-naïve patients who have not received therapy. This heterogeneity, even before therapy, may explain why serum levels do not always reflect tumor burden or treatment efficacy.

The lifespan of CTCs in peripheral blood is estimated at 1 to 2.4 hours (41, 42). CTCs are constantly replenished and hence the DLA procedure does not affect the CTC counts in peripheral blood (43). Given that the PSA half-life is 2.6 days, exploring how the serum levels accumulate and how much the CTC contributes throughout its lifespan is intriguing. It has been previously established that the CTC and PSA levels do not always correlate with each other (44), as also described here. The nanowell array platform used in this study allows for quantifying PSA secreted by each cell present in a microwell. The average secretion of PSA ranged from 4 to 11.68 pg/cell/day (mean, 6.38 ± 2.29). The PSA derived from CTCs and the PSA serum levels show a weak positive correlation with Spearman coefficient ρ = 0.28; however, further investigation using a larger patient cohort is required to improve this relationship. Of the CTCs captured on the nanowells from the 15 patients, the contribution of PSA-secreting cells was observed to be an average of only 31%. An interesting observation is that we identified two subpopulations of secreting cells, namely the PSMA^+^ (mean, 23%) and PSMA^−^ cells (mean, 8%), which may or may not be CK^+^. Although this confirms the presence of prostate cancer cells that do not express PSMA at a level that could be detected, it also raises the question of whether the EpCAM^+^ calcein^+^ CD45^−^ cells present in the microwell array that did not produce PSA are of tumor origin and this warrants further investigation.

Apart from the morphologic differences in secreting cells, we also measured heterogeneous secretion patterns in five of the 18 patients for whom sufficient CTCs were captured in the nanowells. A population of high, medium, and low secretors at varying mean intensity levels was observed, as indicated in Fig. 5D (nanowells 1, 2, and 3) and Fig. 6. The regulation of PSA from CTCs may be influenced by multiple factors. This heterogeneity observed in PSA secretion can be attributed to the differences in cell cycle phases of the CTCs. Studies have explored the effect of androgen suppression via a G_1_ phase arrest of the cell cycle, leading to a decrease in PSA secretion from the cell lines, thereby indicating that PSA production is dependent on cell cycle (45, 46). As PSA is driven by the AR signaling, a possible impairment in this signaling due to the differential AR expression and presence of splice variants, indicating a more aggressive disease (47), could lead to the heterogeneity in secretion. The non-secreting cells that account for an average of 69% in the 15 patients with mCNPC was a surprising finding, as these patients have not been treated with any therapy, not even ADT. Neuroendocrine differentiation (NED) is frequently observed in pretreated patients with mCNPC and metastatic CRPC in whom the CTCs characteristically neither secrete PSA nor express PSMA (48–50). Although the patients in this study did not receive any prior treatment, the presence of PSMA^+^ or PSMA^−^ non-secreting cells may suggest an increase in NED, potentially reflecting a more aggressive CTC phenotype with less AR dependency. However, this interpretation of PSA regulation in CTC remains speculative and warrants further investigation, including the incorporation of molecular analysis of AR signaling, cell cycle markers, and NED-specific markers, such as chromogranin A, to confirm these findings.

The methodology developed in this study opens multiple avenues for translational research apart from PSA quantification. By capturing viable CTCs, this platform can be extended to analyze other secretome markers in real time such as chromogranin A and cancer-specific proteases and cytokines, thereby allowing functional profiling and identification of phenotypic groups contributing to tumor heterogeneity. The platform also allows for drug testing on a single-cell level. Changes in PSA secretion or other secretions from a single CTC in response to ARSI agents, chemotherapy, or immunotherpay agents could serve as a functional readout for drug efficacy. Furthermore, subclonal populations that show changes in secretion, express NED markers, or upregulate immune checkpoint proteins, such as PD-L1 (51), may represent treatment-resistant clones. These CTC clones can be punched for downstream molecular analysis of their nucleic acid composition to further identify genetic aberrations that contribute to treatment resistance. Additionally, high- and low-secreting CTCs can be isolated similarly to develop patient-derived cell lines, organoids, and xenografts. These models can be developed to explore how diverse phenotypic properties correlate with treatment responsiveness and can be used to evaluate therapeutic strategies in a preclinical setting. It is well known that CTCs evolve in their characteristics during their journey from the primary site into circulation in the blood and developing near and distant metastatic foci (52, 53). Cells isolated from resected tumors can be processed similarly to obtain single cells, which can be compared with CTCs to analyze the molecular changes in tumor cells. This approach also provides valuable insights into the contribution of PSA from tumor sites in contrast to the CTC-derived PSA. Lastly, this platform holds potential for biomarker development. Although PSMA expression and PSA secretion have been studied independently, few have investigated the relationship between the two under therapeutic pressure. Mathy and colleagues (54) indicated that PSMA expression and PSA secretion show opposite responses to abiraterone on cell lines. Another study within a cohort of patients with CRPC showed discordance between the PSA kinetics and the PSMA-PET measurements performed, owing to a heterogeneous response to the ARSI agents used (55). In the CNPC setting, a decrease in PSA levels and PSMA expression was observed in response to ADT; however, heterogeneity in PSA response was also observed in a few patients leading to biochemical recurrence (56). Simultaneous assessment of PSA secretion and surface markers such as PSMA allows for the development of an improved composite biomarker, which in combination with the molecular characterization could enhance patient stratification, treatment monitoring, and personalized therapy selection in prostate cancer.

Our study, however, poses a few limitations. Although DLA enrichment significantly improves the number of CTCs that can be characterized, we observed that patients with fewer than 100 CTC counts in peripheral blood were not ideal candidates for measuring PSA secretions in CTCs. Improvement in the capture of CTCs in the nanowells which in this study was only 0% to 25% can significantly improve the number of CTC from which the PSA secretion can be measured. Although the PSMA^−^ cells that secrete PSA in the microwells are likely tumor cells, one cannot be certain of the origin of the PSMA^−^ cells that did not secrete PSA. This can be addressed by additional markers, such as chromogranin A (49, 57, 58) and the malaria protein VAR2CSA (rVAR2; refs. 59–61), to ensure that the EpCAM-enriched sorted (CD45^−^ calcein^+^) cells are of tumor origin. Given the elaborate workflow of the enrichment and sorting of CTCs, followed by capture in the isolated environment in the array, the CTCs may become stressed, leading to apoptosis or possibly necrosis and release of intercellular PSA. The identification of apoptotic markers such as cytochrome C, which is released during apoptosis, can help differentiate the cells that actively secrete from those that release PSA because of death. Another limitation is that the current study evaluated patients with likely poorer prognosis because they were selected based on CTC counts, which means that the observed results may not be reflective of the whole mCNPC group. Larger studies with diverse patient cohorts will be needed to validate and expand on these findings.

### Conclusion

In this study, we present a novel approach to capture and measure PSA secretion from a viable single CTC from the DLA of patients with mCNPC who had not received any prior therapy (including ADT). The viable CTCs isolated onto a nanowell array were characterized as PSMA± secreting and PSMA± non-secreting cells. For the first time, we estimated PSA secretions from a single viable CTC, which were observed to be heterogeneous and ranged from an average of 4 to 11.68 pg/cell. We demonstrate that a majority of the CTCs (69%) do not secrete any PSA, highlighting why PSA does not consistently reflect tumor burden or treatment response. This underscores the imperfections of PSA as a standalone biomarker and supports the need for complementary markers. Future studies incorporating molecular analysis are required to further explore and validate the potential mechanisms such as, AR signaling, cell cycle state, and NED, that may regulate this heterogeneity in secretion.

## Supplementary Material

Table S1 Adjusted prostate cancer organoid medium (APCOM) composition

Table S2 CTC recovery after sorting and seeding on nanowells

Figure S1 PSMA positivity was observed to be highest in LNCaP with 88% above the level of 200 and CK positivity of 100% above the level of 50. The cells of both PC3 and RWPE-1 (negative for PSMA) showed 0.9% positivity above 200 for PSMA and 100% CK positivity above 50 for CK.

Figure S2 DLA samples isolated in nanowells (A): Example of a non-sorted DLA sample showing the presence of cell debris with non-specific staining in PE, FITC and APC channels, making it difficult to identify intact live CTC. (B): Example of sorted DLA sample with the absence of cell debris and a clear indication of live CTC in the nanowells.

Figure S3 Visualization of the nanowells and membrane using the VyCAP SPOT software (A) Imprint of IgG antibody printed onto a PVDF membrane using a nanowell array, is used to align and match the secreted proteins to the corresponding cell inside a well. (B) The PSA proteins secreted by the individual CTC is printed onto PVDF membrane. (C) A brightfield image of the nanowell arrays filled with CTC. (D)Viable CTC inside individual wells of the chip. (E) PSMA-PE expression of the CTC inside the nanowells. (F) CD45-APC expression of the CTC inside the chip. Images in the lower left corner illustrate a small part of the membrane showing the IgG spots (A) and PSA spots (B) with their corresponding well-id analyzed in the VyCAP SPOT software. The corresponding CTC secreting PSA can now be located inside the nanowell array (C-F) to determine cell phenotype and isolated for downstream analysis.

Figure S4 PSA protein calibration curve (A): Serial dilutions of PSA protein spotted on PVDF membrane, stained and visualized in FITC. (B): Calibration graph of PSA (pg/μm2) versus the normalized mean intensity of the spots (R2 = 0.97).

Figure S5 Steps used to determine the area of the PSA spots produced by single CTC. Images of the membranes with the PSA products from single CTC cells were segmented in Image J software (version 1.54g). Once the spots were segmented, the minimum and maximum pixel area size was set to exclude artefacts. As the PSA spots were round in shape, a roundness values between 0.64 and 1.0 was used to exclude unwanted objects. (A) depicts the a part of a PVDF membrane with the PSA spots from CTC. (B) Illustrates the segmented image and the area-id. (C) shows a mask generated with Image J used to check which spots were measured. (D) Once the areas of the PSA spots were determined the spot software was used to link the PSA spots, the area of the spots extracted with image J with the corresponding CTC (well-id). In practice, once the correct threshold value and particle size range was determined, the particle analysis plugin in image J was then used to determine the area of each segmented spot. Next, to corelate the spot area and the well-number (the cell that secreted the PSA), the PSA membrane (A), and the segmented membrane (B) were loaded and aligned in the VyCAP spot software. The VyCAP software segments the image to extract the spot Intensity and well-id (PSA membrane (A) and this is correlated with the spot area-id image created in image J. The blue and red box (first row) in panel D, show the well-id and the spot intensity.

Figure S6 Correlation of PSA CTC vs PSA serum in the processed blood volume of 9 mCNPC patients in whom PSA-secreting CTC were identified. A weak positive correlation was observed with Spearman coefficient (ρ) = 0.28.
