# Supplementary material for: PSA Secretion from Single Circulating Tumor Cells of Metastatic Castration-Naïve Prostate Cancer Patients
Source: Cancer Res Commun. 2025 Aug 18;5(8):1359–71. doi: 10.1158/2767-9764.CRC-25-0158 (PMC12358827; doi:10.1158/2767-9764.CRC-25-0158)
Supplement: Table S1 — Adjusted prostate cancer organoid medium (APCOM) composition [file crc-25-0158_table_s1_suppst1.pdf]

Supplementary Table S1: Adjusted prostate cancer organoid medium (APCOM) composition

Reagent	Concentration in media	Stock concentration	Supplier (info supplier, catologue number)
Advanced DMEM/F12[1] (AdMem)	-	-	ThermoFisher Scientific (Waltham, Massechusetts, USA, 12634028)
Hepes[1]	10 mM	1M	ThermoFisher Scientific (15630056)
L-Glutamine[1]	2 mM	200 mM	Lonza (Basel, Swizerland, 17-605E)
Penicilline/streptomycine[1]	100 U and 100 µg/mL	10.000 U and 10.000 µg/mL	Lonza (17-602E)
Noggin	0.1 µg/ml	100 µg /ml	PeproTech (Rocky Hill, New Jersey, USA, 100-26)
R-spondin	0.5 µg/ml	500ug/ml	PeproTech (Rocky Hill, New Jersey, USA, 100-26)
Epithelial growth factor (EGF)	20 ng/mL	10 µg/mL	Sigma-Aldrich (Saint Louis, Missouri, USA, F29644)
Fibroblast growth factor 2 (FGF-2)	5 ng/mL	50 µg/mL	R&D Systems (Minneapolis, Minnesota, USA, 233-FB-025)
Fibroblast growth factor 10 (FGF-10)	10 ng/mL	100 µg/mL	PeproTech (Rocky Hill, New Jersey, USA, 100-26)
A-83-01	500 nM	25 mM	Tocris Bioscience (Bristol, UK, 2939)
Prostaglandin E2 (PGE2)	1 µM	10 mM	Tocris Bioscience (2296)
Y-27632 dihydrochloride	10 µM	10 mM	Abmole Bioscience (Houston, Texas, USA,M1817)
R1881	5nM	20mM	Biotang Inc., Lexington, MA, USA
[1] Components for AdMem^{+++}. APCOM additives are diluted in AdMem^{+++} and top up till desired volume.			
