# Supplementary material for: PSA Secretion from Single Circulating Tumor Cells of Metastatic Castration-Naïve Prostate Cancer Patients
Source: Cancer Res Commun. 2025 Aug 18;5(8):1359–71. doi: 10.1158/2767-9764.CRC-25-0158 (PMC12358827; doi:10.1158/2767-9764.CRC-25-0158)
Supplement: Table S2 — CTC recovery after sorting and seeding on nanowells [file crc-25-0158_table_s2_suppst2.pdf]

Supplementary Table S2: CTC recovery after sorting and seeding on nanowells

Patient DLA	CTC in 7.5ml of blood	Sorted Calcein+ CD45- cells (input 200 * 10^6)	Captured CTC in nanowells
1	3	1356	0
2	9	1081	0
3	4	353	0
4	10	544	2
5	24	967	5
6	8	46	8
7	4	1509	4
8	9	1477	3
9	46	93	6
10	16	140	1
11	190	288	7
12	4	2560	4
13	4	2136	5
14	63	300	17
15	145	2079	55
16	467	2656	69
17	615	4812	238
18	816	7046	1756
