# Supplementary material for: PSA Secretion from Single Circulating Tumor Cells of Metastatic Castration-Naïve Prostate Cancer Patients
Source: Cancer Res Commun. 2025 Aug 18;5(8):1359–71. doi: 10.1158/2767-9764.CRC-25-0158 (PMC12358827; doi:10.1158/2767-9764.CRC-25-0158)
Supplement: Figure S1 — PSMA positivity was observed to be highest in LNCaP with 88% above the level of 200 and CK positivity of 100% above the level of 50. The cells of both PC3 and RWPE-1 (negative for PSMA) showed 0.9% positivity above 200 for PSMA and 100% CK positivity above 50 for CK. [file crc-25-0158_figure_s1_suppsf1.pdf]

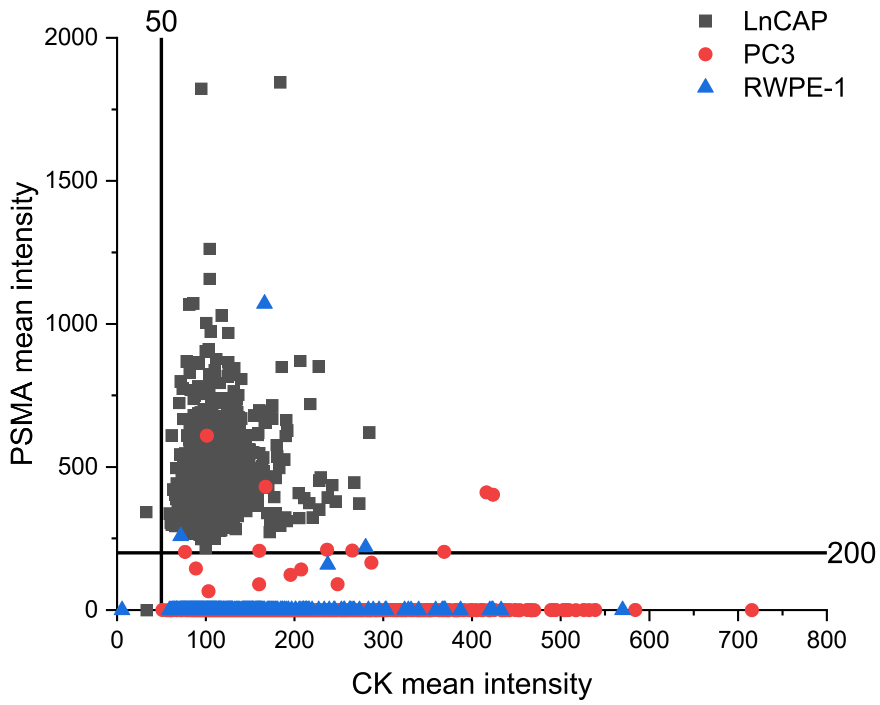

Supplementary Figure S1: PSMA positivity was observed to be highest in LNCaP with 88% above the level of 200 and CK positivity of 100% above the level of 50. The cells of both PC3 and RWPE-1 (negative for PSMA) showed 0.9% positivity above 200 for PSMA and 100% CK positivity above 50 for CK.
