# Supplementary material for: PSA Secretion from Single Circulating Tumor Cells of Metastatic Castration-Naïve Prostate Cancer Patients
Source: Cancer Res Commun. 2025 Aug 18;5(8):1359–71. doi: 10.1158/2767-9764.CRC-25-0158 (PMC12358827; doi:10.1158/2767-9764.CRC-25-0158)
Supplement: Figure S2 — DLA samples isolated in nanowells (A): Example of a non-sorted DLA sample showing the presence of cell debris with non-specific staining in PE, FITC and APC channels, making it difficult to identify intact live CTC. (B): Example of sorted DLA sample with the absence of cell debris and a clear indication of live CTC in the nanowells. [file crc-25-0158_figure_s2_suppsf2.pdf]

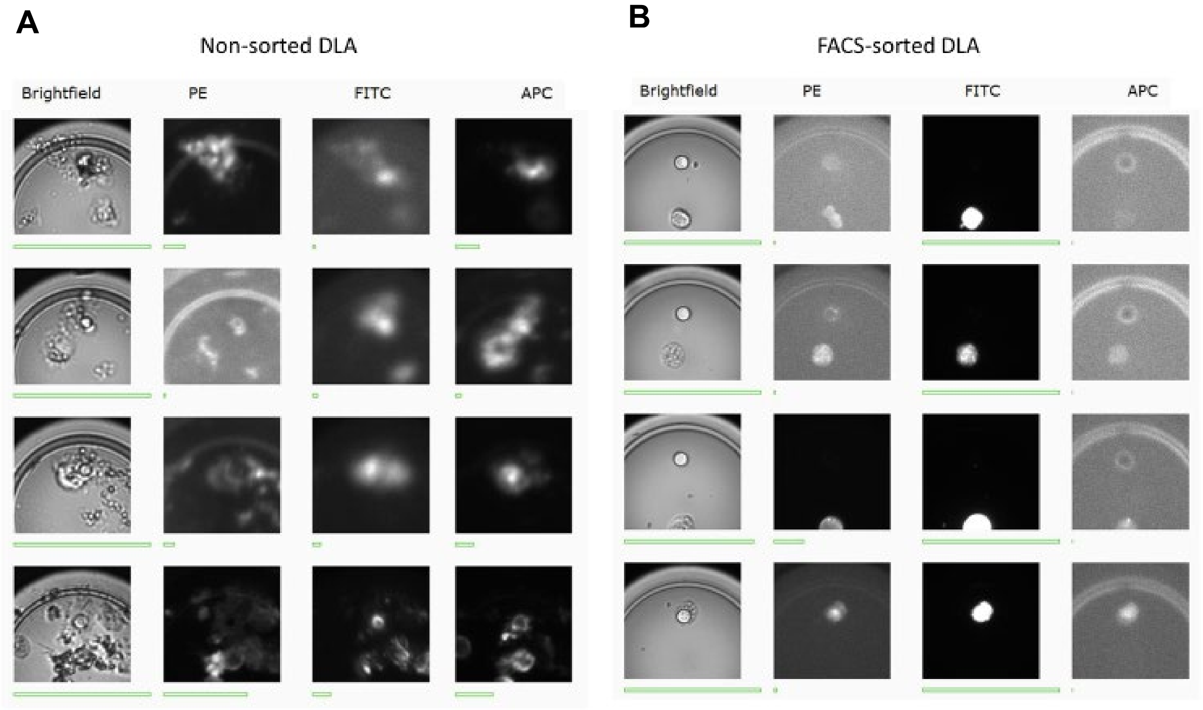

Supplementary Figure S2: DLA samples isolated in nanowells **(A):** Example of a non-sorted DLA sample showing the presence of cell debris with non-specific staining in PE, FITC and APC channels, making it difficult to identify intact live CTC. **(B):** Example of sorted DLA sample with the absence of cell debris and a clear indication of live CTC in the nanowells.
