# Supplementary material for: PSA Secretion from Single Circulating Tumor Cells of Metastatic Castration-Naïve Prostate Cancer Patients
Source: Cancer Res Commun. 2025 Aug 18;5(8):1359–71. doi: 10.1158/2767-9764.CRC-25-0158 (PMC12358827; doi:10.1158/2767-9764.CRC-25-0158)
Supplement: Figure S3 — Visualization of the nanowells and membrane using the VyCAP SPOT software (A) Imprint of IgG antibody printed onto a PVDF membrane using a nanowell array, is used to align and match the secreted proteins to the corresponding cell inside a well. (B) The PSA proteins secreted by the individual CTC is printed onto PVDF membrane. (C) A brightfield image of the nanowell arrays filled with CTC. (D)Viable CTC inside individual wells of the chip. (E) PSMA-PE expression of the CTC inside the nanowells. (F) CD45-APC expression of the CTC inside the chip. Images in the lower left corner illustrate a small part of the membrane showing the IgG spots (A) and PSA spots (B) with their corresponding well-id analyzed in the VyCAP SPOT software. The corresponding CTC secreting PSA can now be located inside the nanowell array (C-F) to determine cell phenotype and isolated for downstream analysis. [file crc-25-0158_figure_s3_suppsf3.pdf]

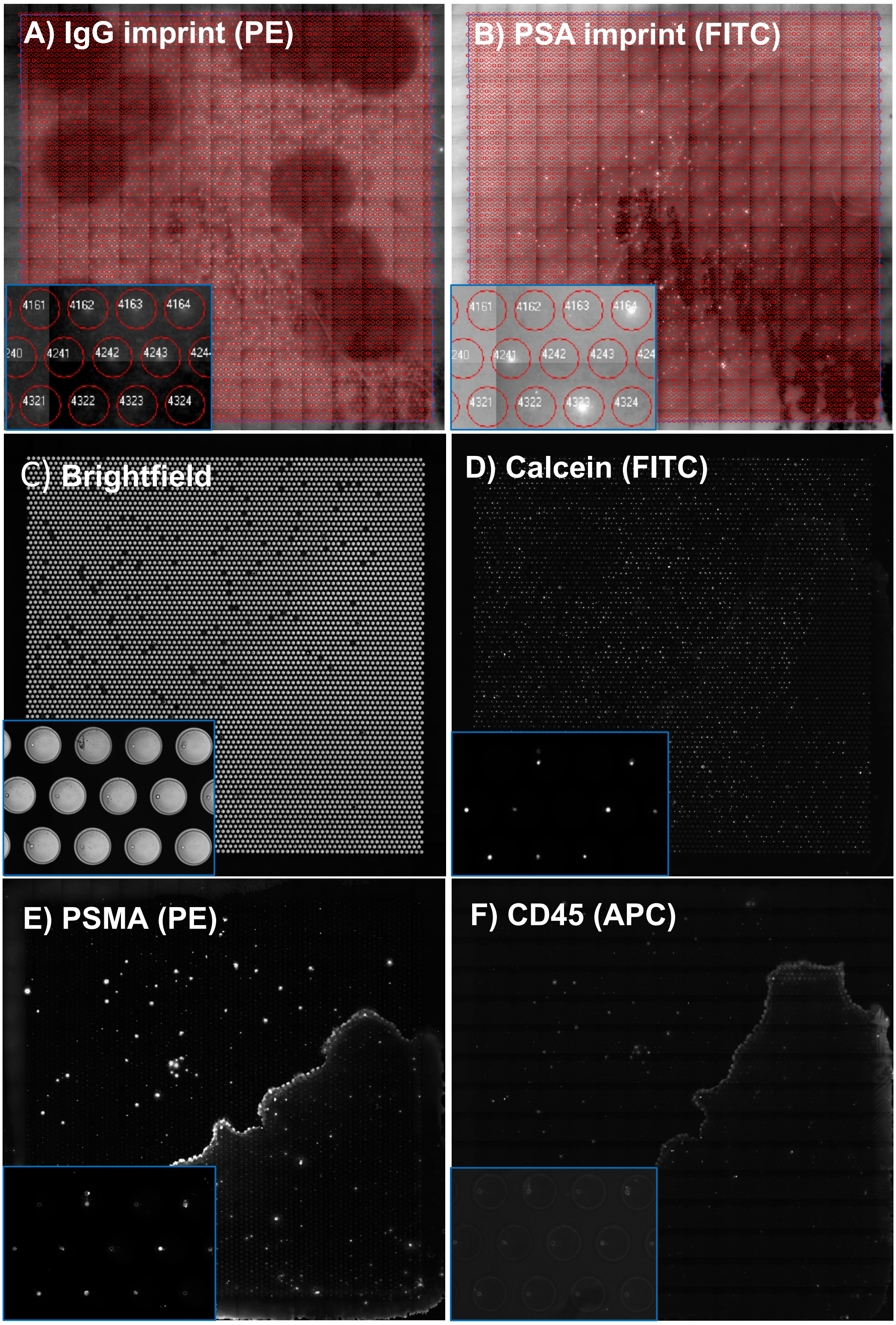


**Supplementary Figure S3:** Visualization of the nanowells and membrane using the VyCAP SPOT software **(A)** Imprint of IgG antibody printed onto a PVDF membrane using a nanowell array, is used to align and match the secreted proteins to the corresponding cell inside a well. **(B)** The PSA proteins secreted by the individual CTC is printed onto PVDF membrane. **(C)** A brightfield image of the nanowell arrays filled with CTC. **(D)**Viable CTC inside individual wells of the chip. **(E)** PSMA-PE expression of the CTC inside the nanowells. **(F)** CD45-APC expression of the CTC inside the chip. Images in the lower left corner illustrate a small part of the membrane showing the IgG spots (A) and PSA spots (B) with their corresponding well-id analyzed in the VyCAP SPOT software. The corresponding CTC secreting PSA can now be located inside the nanowell array (C-F) to determine cell phenotype and isolated for downstream analysis.
