# Supplementary material for: PSA Secretion from Single Circulating Tumor Cells of Metastatic Castration-Naïve Prostate Cancer Patients
Source: Cancer Res Commun. 2025 Aug 18;5(8):1359–71. doi: 10.1158/2767-9764.CRC-25-0158 (PMC12358827; doi:10.1158/2767-9764.CRC-25-0158)
Supplement: Figure S4 — PSA protein calibration curve (A): Serial dilutions of PSA protein spotted on PVDF membrane, stained and visualized in FITC. (B): Calibration graph of PSA (pg/μm2) versus the normalized mean intensity of the spots (R2 = 0.97). [file crc-25-0158_figure_s4_suppsf4.pdf]

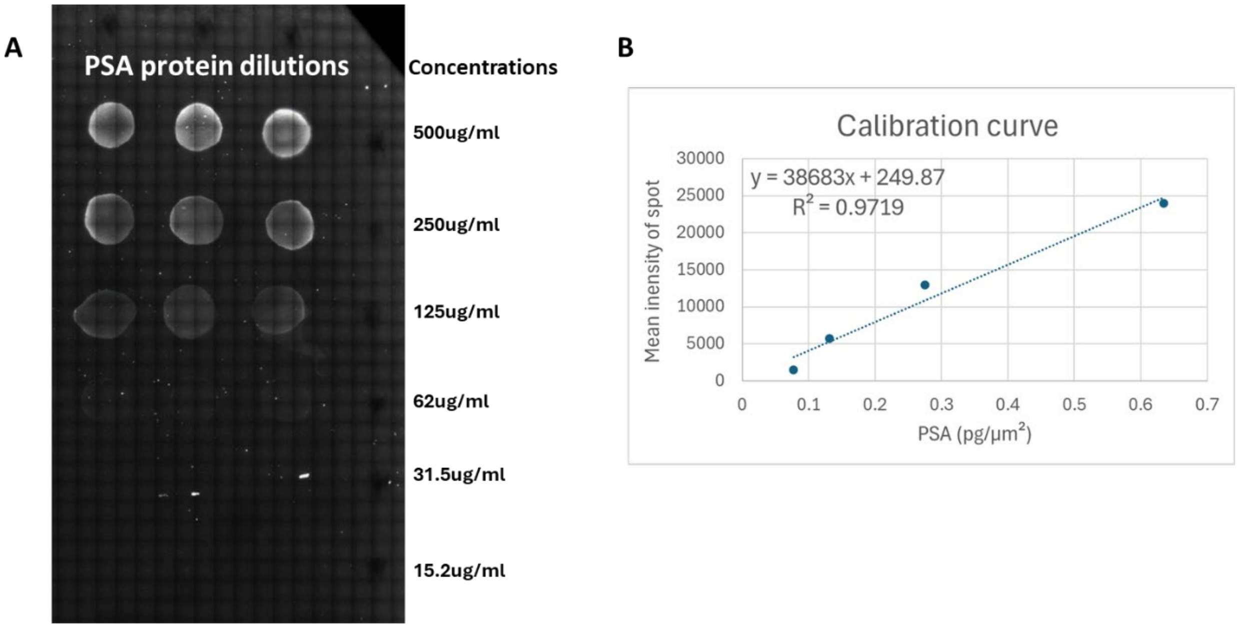

Supplementary Figure S4: PSA protein calibration curve (**A): ** Serial dilutions of PSA protein spotted on PVDF membrane, stained and visualized in FITC. **(B):** Calibration graph of PSA (pg/μm^2) versus the normalized mean intensity of the spots (R^2 = 0.97).
