# Supplementary material for: PSA Secretion from Single Circulating Tumor Cells of Metastatic Castration-Naïve Prostate Cancer Patients
Source: Cancer Res Commun. 2025 Aug 18;5(8):1359–71. doi: 10.1158/2767-9764.CRC-25-0158 (PMC12358827; doi:10.1158/2767-9764.CRC-25-0158)
Supplement: Figure S5 — Steps used to determine the area of the PSA spots produced by single CTC. Images of the membranes with the PSA products from single CTC cells were segmented in Image J software (version 1.54g). Once the spots were segmented, the minimum and maximum pixel area size was set to exclude artefacts. As the PSA spots were round in shape, a roundness values between 0.64 and 1.0 was used to exclude unwanted objects. (A) depicts the a part of a PVDF membrane with the PSA spots from CTC. (B) Illustrates the segmented image and the area-id. (C) shows a mask generated with Image J used to check which spots were measured. (D) Once the areas of the PSA spots were determined the spot software was used to link the PSA spots, the area of the spots extracted with image J with the corresponding CTC (well-id). In practice, once the correct threshold value and particle size range was determined, the particle analysis plugin in image J was then used to determine the area of each segmented spot. Next, to corelate the spot area and the well-number (the cell that secreted the PSA), the PSA membrane (A), and the segmented membrane (B) were loaded and aligned in the VyCAP spot software. The VyCAP software segments the image to extract the spot Intensity and well-id (PSA membrane (A) and this is correlated with the spot area-id image created in image J. The blue and red box (first row) in panel D, show the well-id and the spot intensity. [file crc-25-0158_figure_s5_suppsf5.pdf]

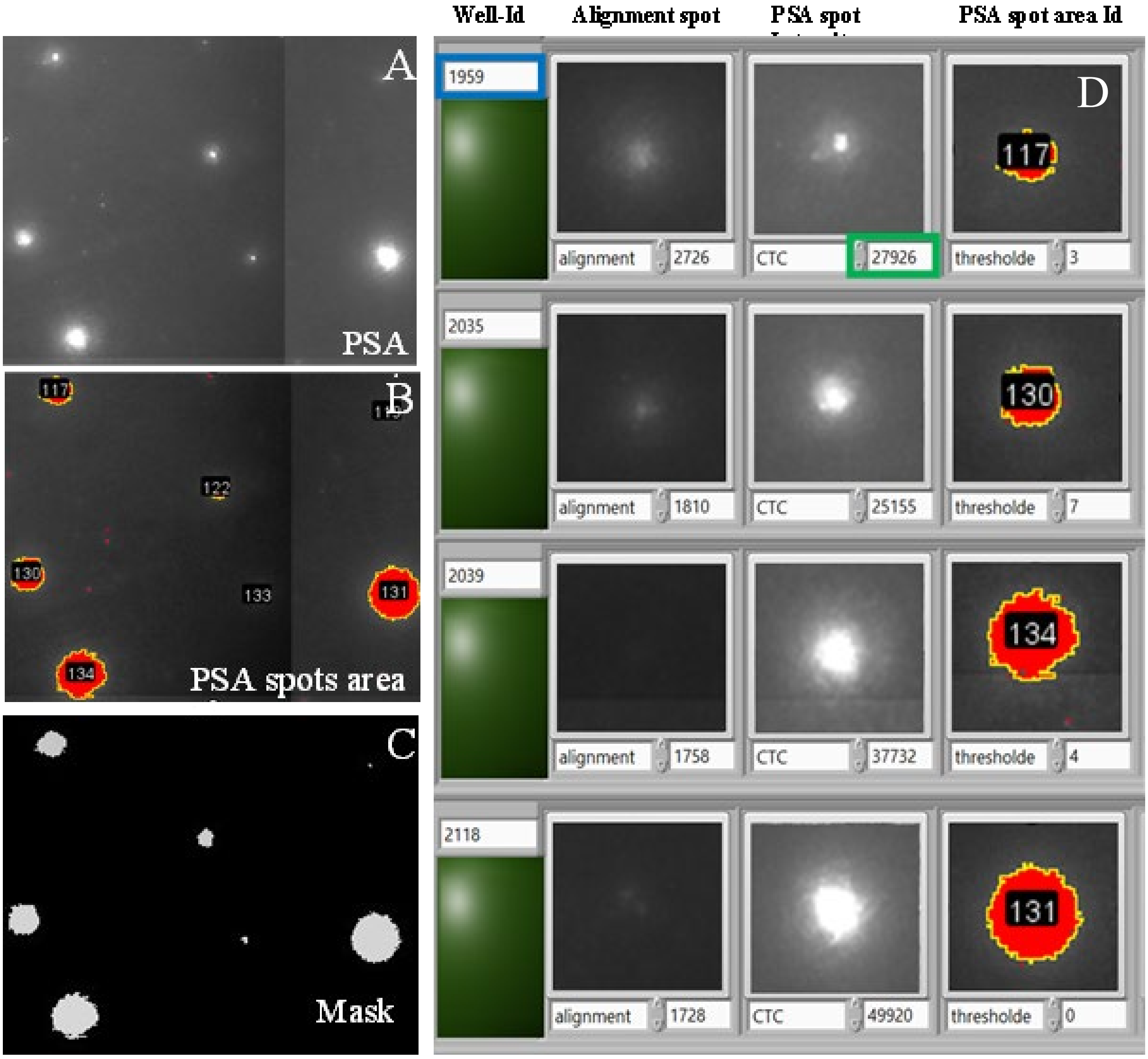

Supplementary Figure S5: Steps used to determine the area of the PSA spots produced by single CTC. Images of the membranes with the PSA products from single CTC cells were segmented in Image J software (version 1.54g). Once the spots were segmented, the minimum and maximum pixel area size was set to exclude artefacts. As the PSA spots were round in shape, a roundness values between 0.64 and 1.0 was used to exclude unwanted objects. **(A)** depicts the a part of a PVDF membrane with the PSA spots from CTC. **(B)** Illustrates the segmented image and the area-id. **(C)** shows a mask generated with Image J used to check which spots were measured. **(D)** Once the areas of the PSA spots were determined the spot software was used to link the PSA spots, the area of the spots extracted with image J with the corresponding CTC (well-id). In practice, once the correct threshold value and particle size range was determined, the particle analysis plugin in image J was then used to determine the area of each segmented spot. Next, to corelate the spot area and the well-number (the cell that secreted the PSA), the PSA membrane (A), and the segmented membrane (B) were loaded and aligned in the VyCAP spot software. The VyCAP software segments the image to extract the spot Intensity and well-id (PSA membrane (A) and this is correlated with the spot area-id image created in image J. The blue and red box (first row) in panel D, show the well-id and the spot intensity.
