# Supplementary material for: PSA Secretion from Single Circulating Tumor Cells of Metastatic Castration-Naïve Prostate Cancer Patients
Source: Cancer Res Commun. 2025 Aug 18;5(8):1359–71. doi: 10.1158/2767-9764.CRC-25-0158 (PMC12358827; doi:10.1158/2767-9764.CRC-25-0158)
Supplement: Figure S6 — Correlation of PSA CTC vs PSA serum in the processed blood volume of 9 mCNPC patients in whom PSA-secreting CTC were identified. A weak positive correlation was observed with Spearman coefficient (ρ) = 0.28. [file crc-25-0158_figure_s6_suppsf6.pdf]

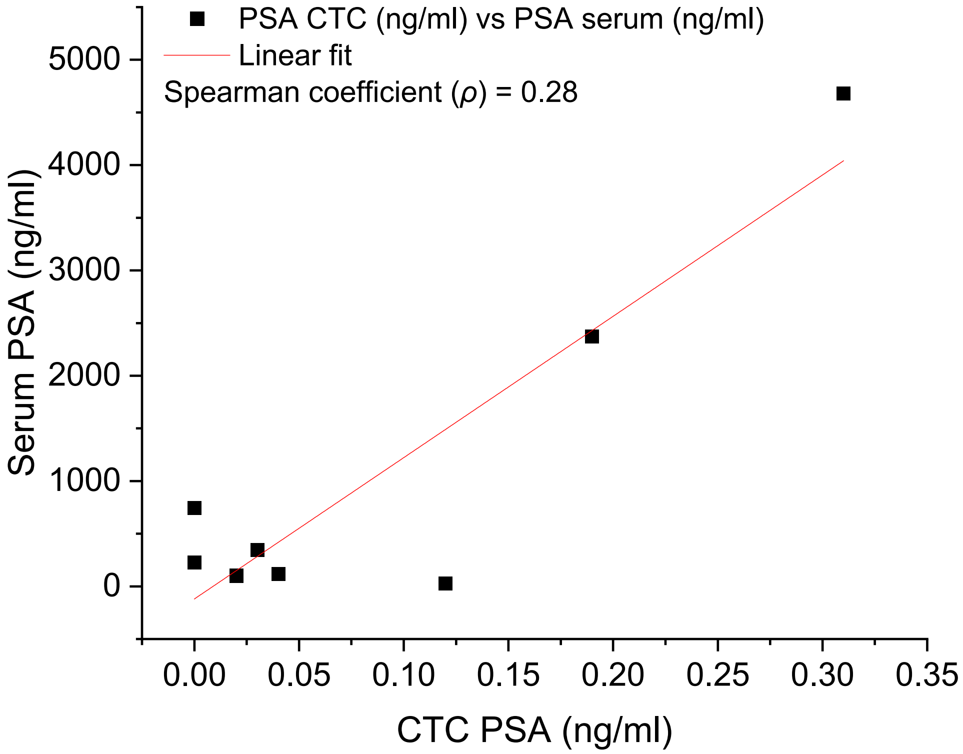

Supplementary Figure S6: Correlation of PSA CTC vs PSA serum in the processed blood volume of 9 mCNPC patients in whom PSA-secreting CTC were identified. A weak positive correlation was observed with Spearman coefficient (ρ) = 0.28.
